# Prevalence and Genetic Diversity of *Haemoproteus* and *Leucocytozoon* in Raptors and Other Captive Birds at the National Zoological Garden in South Africa

**DOI:** 10.1111/1749-4877.70011

**Published:** 2025-10-29

**Authors:** Realeboga Masego Gaorekwe, Veronica Phetla, Dikeledi Petunia Malatji, Mamohale Chaisi

**Affiliations:** ^1^ Foundational Biodiversity Science South African National Biodiversity Institute Pretoria South Africa; ^2^ Department of Agriculture and Animal Health University of South Africa Roodepoort South Africa; ^3^ Department of Veterinary Tropical Diseases University of Pretoria Onderstepoort South Africa

**Keywords:** avian haemosporidia, captive birds, *Haemoproteus* spp, *Leucocytozoon* spp, South Africa

## Abstract

Avian haemosporidian infections have been associated with disease outbreaks in zoos and rehabilitation centers globally. This study aimed to determine the occurrence and genetic diversity of avian haemosporidian parasites in captive birds at the National Zoological Garden in South Africa. One hundred and eighty‐three blood samples from five orders and 15 species of captive flamingos, vultures, owls, ibises and parrots were analyzed for haemosporidia by nested polymerase chain reaction assays. The samples were collected as part of the zoo's studbook and archived at South African National Biodiversity Institute's Wildlife Biobank. The overall infection rate was 36.1%, and infections by *Leucocytozoon* spp. (33.3%) were significantly higher than *Haemoproteus* spp. (14.8%) (*p* < 0.001). Twenty‐one samples (11.5%) had mixed *Haemoproteus* and *Leucocytozoon* infections. The Spotted Eagle Owl *(Bubo africanus*) and Barn Owl (*Tyto alba*) had the highest infection rates. Twenty‐six sequences, similar to published sequences of *Leucocytozoon* spp. (lineages lCHRKLA02, lCIAE02, and lBUVIR02) and *Haemoproteus* spp. (lineage hTYTAL6) were obtained. Two new lineages (lBUBCAP01 and hBOSHAG02) are described in this study. This is the first molecular survey of haemosporidian parasites in captive birds of the orders Accipitriformes, Pelecaniformes, Psittaciformes, Phoenicopteriformes, and Strigiformes in South Africa. This study provides new geographical and host records of known and novel haemosporidian lineages. It highlights the need for intensive surveys of these parasites in populations of captive and free‐ranging birds in South Africa, regular monitoring of infections, updated screening methods, and insect control in the zoo's animal collection to avoid disease outbreaks.

## Graphical abstract

Captive birds at the National Zoological Garden in South Africa harbor diverse haemosporidian parasite species and lineages. This might compromise conservation efforts of threatened species.

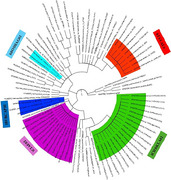

## 1 Introduction

Haemosporidian parasites of the genera *Plasmodium*, *Haemoproteus*, and *Leucocytozoon* (Phylum: Apicomplexa; Order Haemosporida) have been identified in reptiles, birds, and mammals worldwide (Levine 1988; Harl et al. 2020). Mosquitoes (mainly *Culex* spp.) transmit *Plasmodium* spp. The genus *Haemoproteus* comprises two subgenera, namely, the genus *Haemoproteus*, transmitted by hippoboscid (louse) flies, and the genus *Parahaemoproteus*, transmitted by *Culicoides* (biting midges), while *Leucocytozoon* species are transmitted by simulium flies (Valkiūnas 2005; Valkiūnas et al. 2008; Ranford‐Cartwright 2023). According to the MalAvi database (Bensch et al. 2009), over 250 species of avian haemosporidia (*Haemoproteus, Plasmodium*, and *Leucocytozoon* spp.) have been described based on morphology. Molecular data of partial *cytochrome b* (*cyt b*) gene fragments of these parasites indicate much greater diversity. However, most of these lineages are still not described at the species level. Infections in birds can result in severe disease or death of native bird populations, depending on the parasite species and lineage, and the age and immune status of the host (Niebuhr et al. 2016; Palinauskas et al. 2016). Juveniles and non‐adapted birds are susceptible to infection, often resulting in death after their first exposure to infection (Atkinson 2005; Jia et al. 2018).

Free‐ranging and migratory birds usually have a heavy parasite load and diverse haemosporidian species or lineages during the short peak phases of infections and may act as a source of infection to captive and domestic birds (Botes et al. 2017; Chaisi et al. 2019; Meister et al. 2023). Migratory birds are more exposed to infection than resident birds as they travel across diverse regions (de Angeli Dutra et al. 2021). Previous studies indicate that birds in captivity are prone to infection by haemosporidian parasites, and disease outbreaks in captive birds are common (Alley et al. 2008; Grilo et al. 2016; Chagas et al. 2017; Meister et al. 2023). This can compromise the conservation efforts of endangered birds in captive facilities. Fatal haemosporidiosis has been reported from captive birds collected during necroscopy in zoos and other conservation facilities in different parts of the world (Vanstreels et al. 2015; Jia et al. 2018; Galosi et al. 2019; Meister et al. 2023). Acute infections can result in hepatomegaly, splenomegaly, and pulmonary edema and are thought to result from vascular occlusion and rupture of tissue meronts in the cell (Grilo et al. 2016). Regular monitoring of infections in captive birds is therefore vital for the success of conservation or rehabilitation programs in zoos and rehabilitation facilities. Conservation facilities also offer a unique opportunity to study these parasites and determine their burden and impact on bird health (Jia et al. 2018; Himmel et al. 2021), as there is a paucity of information on the effect of avian haemosporidia on the health of the threatened taxa globally.

The National Zoological Garden (NZG), a facility of the South African National Biodiversity Institute (SANBI), is home to approximately 1358 specimens of 202 bird species, among other animals (https://www.pretoriazoo.org/about/). These include native species listed as highly endangered or vulnerable by the International Union for Conservation of Nature's (IUCN) List of Threatened Species (https://www.iucnredlist.org/). As a result, several initiatives at the NZG aim to safeguard these species in their natural habitats in support of national Biodiversity Management Plans, such as the Cape Vulture [*Gyps coprotheres* (Forster, 1798)] breeding program, which has been breeding and releasing Cape Vultures into the wild since 1996 (https://www.pretoriazoo.org/pretoria‐zoos‐cape‐vulture‐breeding‐programme‐is‐flying‐high/). However, the threat of disease may compromise these conservation efforts. Hence, this study aimed to determine the occurrence, diversity, and phylogenetic relationships of haemosporidian parasites of selected species of captive birds at the NZG to inform disease management.

## 2 Materials and Methods

### 2.1 Ethical Clearance

Ethical clearance was granted by the University of South Africa (UNISA) Animal Research Ethics Committee (2019/CAES_AREC/142) and the Animal Research and Scientific Ethics Committee (ARESC) of the SANBI (SANBI/RES/P2021/01). Permission to do research in terms of Section 20 of the Animal Diseases Act, 1984, was obtained from the Department of Agriculture, Land Reform and Rural Development, South Africa, reference number 12/11/1/1/18 (1001).

### 2.2 Study Area and Origin of Samples

The study was conducted at the NZG, South Africa (25°44ʹ10.79ʹʹS, 28°11ʹ12.60ʹʹE). The NZG animal collection includes threatened bird species native to southern Africa. A total of 183 blood samples, collected in EDTA tubes (*n* = 177) and on filter paper (*n* = 6) between April 2006 and May 2015 and archived at the SANBI Wildlife Biobank, were analyzed for the presence of haemosporidian parasites. The samples originated from 15 species of captive birds in five orders, namely, Accipitriformes, Pelecaniformes, Strigiformes, Phoenicopteriformes, and Psittaciformes (Table 1). Blood smears were not available for morphological identification of the parasites.

**TABLE 1:** Prevalence of *Leucocytozoon* spp. and *Haemoproteus* spp. in avian hosts as determined by the nested PCR.

Order	Species (common name)	Authority †	Number of samples	Number of infections			
				*Leucocytozoon*	*Haemoproteus*	Mixed infections	Total (%)
Accipitriformes	*Sarcoramphus papa* (King Vulture)	(Linnaeus, 1758)	8	0	0	0	0
	*Gyps coprotheres* (Cape Vulture)	(Forster, 1798)	20	13	0	0	13 (65%)
Phoenicopteriformes	*Phoenicopterus roseus* (Greater Flamingo)	Pallas, 1811	20	1	1	1	1 (5%)
	*Phoeniconaias minor* (Lesser Flamingo)	(Geoffroy Saint‐Hilaire, 1798)	4	3	0	0	3 (75%)
Strigiformes	*Strix woodfordii* (African Wood Owl)	(Smith, 1834)	4	4	0	0	4 (100%)
	*Tyto alba* (Barn Owl)	(Scopoli, 1769)	13	12	4	4	12 (92%)
	*Bubo capensis* (Cape Eagle Owl)	(Smith, 1834)	8	2	0	0	2 (25%)
	*Bubo africanus* (Spotted Eagle Owl)	(Temminck, 1821)	20	15	14	15	15 (75%)
Pelecaniformes	*Geronticus calvus* (Southern Bald Ibis)	(Boddaert, 1783)	20	0	1	0	1 (5%)
	*Plegadis falcinellus* (Glossy Ibis)	(Linnaeus, 1766)	3	3	0	0	3 (100%)
	*Eudocimus ruber* (Scarlet Ibis)	(Linnaeus, 1758)	20	1	2	1	2 (1%)
	*Bostrychia hagedash* (Hadada Ibis)	(Latham, 1790)	19	3	1	0	3 (2%)
Psittaciformes	*Psittacus erithacus* (African Grey Parrot)	Linnaeus, 1758	20	0	0	0	0
	*Poicephalus meyeri* (Meyer's Parrot)	(Cretzschmar, 1827)	1	1	0	0	1 (100%)
	*Poicephalus robustus* (Cape Parrot)	(Gmelin, 1788)	3	3	0	0	3 (100%)
TOTAL			183	61 (33.3%)	27 (14.8%)	21 (11.5%)	(66) 36.1%

^†^Handbook of the Birds of the World and BirdLife International Digital Checklist of the Birds of the World. Version 9.1 (http://datazone.birdlife.org/species/taxonomy).

### 2.3 DNA Extraction

Genomic DNA was extracted from whole blood using the Zymo Research Quick‐DNA Universal kit, and from blood spots on filter paper using the Zymo Research Genomic DNA Tissue Miniprep kit (Inqaba Biotechnologies, South Africa) according to the manufacturer's instructions. The DNA was eluted with 45 µL DNA Elution Buffer and the DNA concentration was determined by the NanoDrop 1000 spectrophotometer (Thermo Fisher Scientific).

### 2.4 Polymerase Chain Reaction

The *cyt b* gene of *Haemoproteus*, *Plasmodium*, and *Leucocytozoon* was amplified using the commonly used nested polymerase chain reaction (PCR) assays described by Hellgren et al. (2004) and Waldenström et al. (2004). The primary reaction consisted of 2 µL (app. 40 ng) template DNA, 12.5 µL. Ampliqon Red Taq DNA Polymerase Master Mix (Lasec, South Africa), 0.4 µM of primers HaemNF1 and HaemNR3 and molecular‐grade ddH_2_O to a final volume of 25 µL. For the nested PCR, 1 µL of the PCR amplicon was used as a template. Primers HaemF and HaemR2 were used to amplify a 480 bp fragment of *Plasmodium* and *Haemoproteus* spp. and 478 bp of *Leucocytozoon* spp. using primers HaemFL and HaemR2L.

Positive controls consisted of synthetic DNA fragments (gBlocks) from *Plasmodium relictum* and *Leucocytozoon fringillinarum* (GenBank accession# FJ168564), while molecular‐grade water served as the negative control. Both controls were included in each PCR run. The cycling conditions were as indicated by Hellgren et al. (2004) and Waldenström et al. (2004). A volume of 5 µL of the nested PCR products was visualized on 2% agarose gel which was stained with the SYBR Safe DNA Gel Stain (Thermo Fisher Scientific, South Africa). Mixed infections of *Leucocytozoon* and *Haemoproteus*/*Plasmodium* were identified from the nested PCR results by amplicons of the expected fragment sizes (478–480 bp) on two different gels. Amplicons (20 µL) were submitted to Inqaba Biotechnologies (South Africa) for bi‐directional cycle sequencing using the nested PCR primers.

### 2.5 Sequence and Phylogenetic Analyses

The forward and reverse reads were assembled using Geneious (v10.2.6) (http://www.geneious.com, Kearse et al. 2012) and aligned using the MUSCLE alignment tool (Edgar 2004) within Geneious. Consensus sequences were subjected to a Basic Local Alignment Search Tool (BLAST) from the MalAvi database to identify infecting lineages (*cyt b* haplotypes) (Bensch et al. 2009;) and from GenBank (www.ncbi.nlm.nih.gov/genbank/) for the identification of homologous sequences that might be missing from the MalAvi database. Sequences that differ by one or more base pairs (<100% similarity) from known parasite lineages in MalAvi were described as novel lineages, as suggested by Hellgren et al. (2004) and were designated new lineage names. Phylogenetic trees were reconstructed by Maximum Likelihood (ML) using MEGA X (Kumar et al. 2018) and Bayesian Inference (BI) using MrBayes version 2.2.3 (Huelsenbeck and Ronquist 2001) plug‐in in Geneious. The best‐fitting model was N93+G as determined in MEGA X. Bootstrap values were obtained from 1000 pseudoreplicates for the ML tree, and the Markov chain Monte Carlo (MCMC) model was run using 4 chains of 1.1 million generations, with sampling every 500 generations. The first 25% of trees were discarded as “burn‐in,” and the remaining trees were used to construct a consensus tree. The phylogenetic analysis included 26 *cyt b* sequences obtained from this study, and published sequences of Haemosporidia of Accipitriformes, Pelecaniformes, Strigiformes, Phoenicopteriformes, Psittaciformes, and other birds from the MalAvi database (Bensch et al. 2009), including published sequences of *Plasmodium* (6), *Haemoproteus* (25), and *Leucocytozoon* (22) spp. The *cyt b* sequence of *Toxoplasma gondii* was used as the outgroup, with a total of 80 sequences in the final dataset. Sequences of known morphospecies of the three parasite genera were included to possibly identify the new lineages from South Africa. The resulting trees were viewed and edited using MEGAX. The host nomenclature used in this paper is according to the IOC World Bird List (available at https://www.worldbirdnames.org/new/) and the Handbook of the Birds of the World and BirdLife International digital checklist of the Birds of the World—version 9.1 (available at http://datazone.birdlife.org/species/taxonomy).

## 3 Results

### 3.1 Detection of Avian Haemosporidian Parasite Infections by the Nested PCR Assays

The nested PCR detected haemosporidian infections in 66 (36.1%) samples from 13 species of all five orders. Infection ranged from 2% to 100% (Table 1). No infections were detected from two species, namely, the King Vulture [*Sarcoramphus papa* (Linnaeus, 1758)] and the African Grey Parrot [*Psittacus erithacus* (Linnaeus, 1758)]. *Leucocytozoon* spp. infections were identified in 61 (33.3%) samples, and were significantly more common than those of *Haemoproteus* spp., which were identified in 27 (14.8%) samples (*p* < 0.001). Mixed *Leucocytozoon* and *Haemoproteus* spp. infections were identified in 21 (11.5%) birds by nested PCR (Table 1). *Leucocytozoon* spp. were identified in 12 of the 13 infected species of all five orders, except for the Southern Bald Ibis [*Geronticus calvus* (Boddaert, 1783)] and African Grey Parrot (*P. erithacus*); while *Haemoproteus* infections were identified in five species from three orders, namely Greater Flamingo (*Phoenicopterus roseus* Pallas, 1811), Barn Owl [*Tyto alba* (Scopoli, 1769)], Southern Bald Ibis, Scarlet Ibis [*Eudocimus ruber* (Linnaeus, 1758)], and Hadada Ibis [*Bostrychia hagedash* (Latham, 1790)]. All Ibis species, except the Southern Bald Ibis, harbored *Leucocytozoon* infections (Table 1).

### 3.2 Lineage Diversity and Phylogenetic Relationships

**FIGURE 1:**
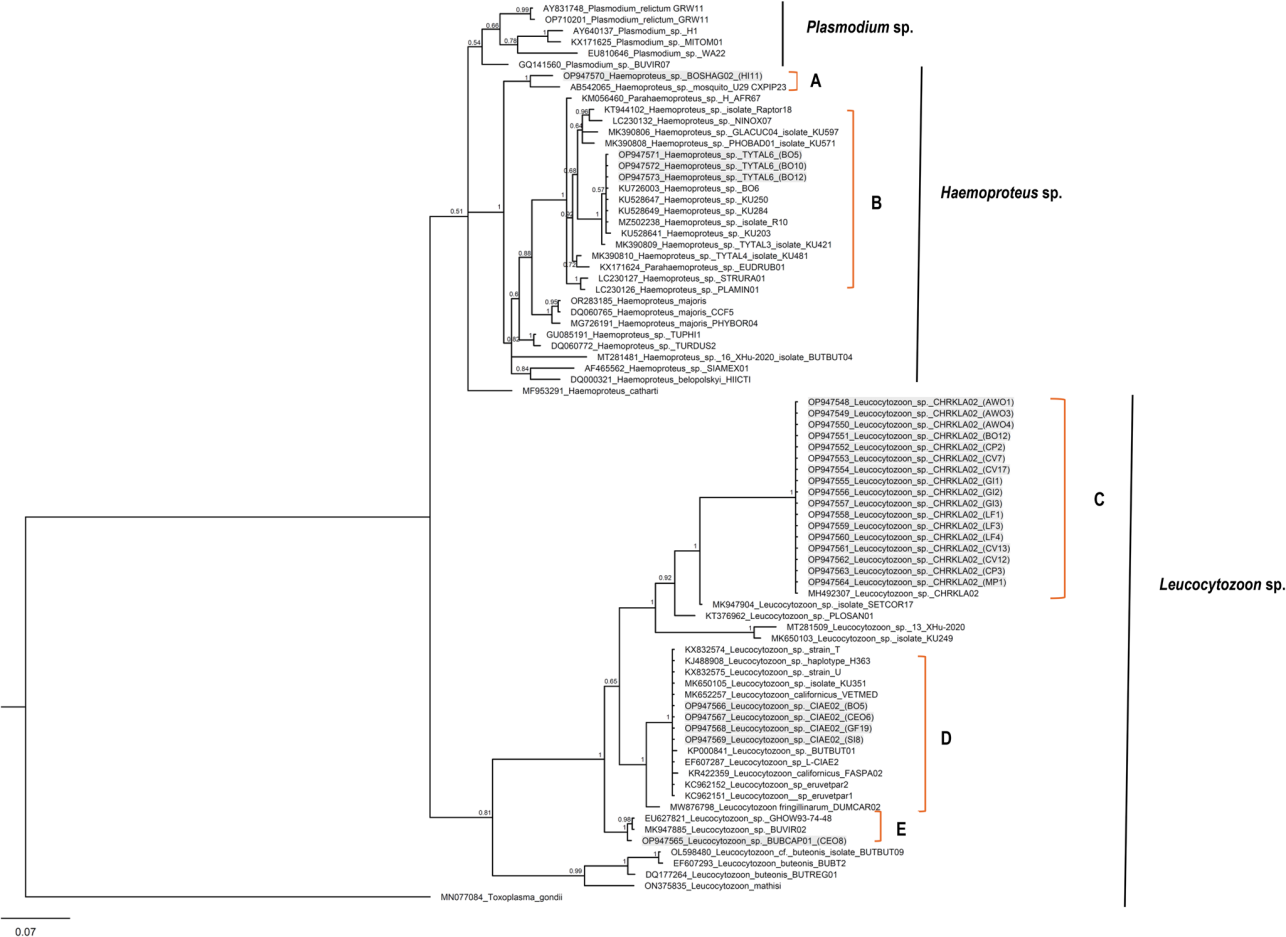
The evolutionary relationships of avian haemosporidian parasites of birds of the orders Accipitriformes (vultures), Pelecaniformes (ibises), Psittaciformes (parrots), Phoenicopteriformes (flamingos), and Strigiformes (owls) based on the partial *cytochrome b* gene (471 base pairs) as inferred by Bayesian analysis. Node values indicate posterior probabilities. GenBank accession numbers and MalAvi lineages, respectively, are indicated before and after the species or genus name. Sequences from this study (Clades A–E) are highlighted in grey, with sample numbers in brackets. *Toxoplasma gondii* was used as the outgroup. Names in brackets indicate sample numbers from this study with prefixes: AW—African Wood Owl; BO—Barn Owl; CE—Cape Eagle Owl; CP—Cape Parrot; CV—Cape Vulture; GF—Greater Flamingo; GI—Glossy Ibis; HI—Hadada Ibis; LF—Lesser Flamingo; MP—Meyer's Parrot; SI—Scarlet Ibis.

We obtained 26 good‐quality sequences from 12 host species. These were 98%–100% similar to published sequences of avian haemosporidia of raptors and other birds from various parts of the world (Table 2). These belonged to five distinct lineages of *Leucocytozoon* and *Haemoproteus* spp., three (lCIAE02, lCHRKLA02, and hTYTAL6) of which are known lineages and have previously been described in vultures and other birds, and two new lineages (lBUBCAP01 and hBOSHAG02) that are described for the first time in this study. Phylogenetic analysis also clustered the *cyt b* sequences (lineages) into five strongly supported clades (A–E; Figure 1). *Leucocytozoon* lineage lCHRKLA02, originally described from Klaas's Cuckoo in South Africa (Chaisi et al. 2019), was the most common lineage identified in this study. Sequences of this lineage were obtained from 17 individuals of seven host species, namely, African Wood Owl, Barn Owl, Cape Parrot, Cape Vulture, Glossy Ibis, Lesser Flamingo, and Meyer's Parrot (Table 2; Figure 1). The second most common lineage (lCIAE02) is a common lineage of different species of bird globally, and was identified from four host species (Barn Owl, Cape Eagle Owl, Greater Flamingo, and Scarlet Ibis). *Haemoproteus* Clade B and *Leucocytozoon* Clade E represent sequences obtained from owls (Strigiformes) and other raptors, while sequences from Clades A, B, and D are from birds in diverse orders and do not demonstrate any host specificity.

**TABLE 2:** *Leucocytozoon* spp. and *Haemoproteus* spp. lineages identified from this study.

		BLAST results					
Parasite genus	Host species	Sample no.	GenBank accession number	MalAvi lineage	Closest match (MalAvi lineage)	GenBank accession number	% Identity
*Leucocytozoon*	*Strix woodfordii*	AWO1	**OP947548**	lCHRKLA02	lCHRKLA02	MH492307	100%
		AWO3	**OP947549**				
		AWO4	**OP947550**				
	*Tyto alba*	BO12	**OP947551**				
	*Poicephalus robustus*	CP2	**OP947552**				
		CP3	**OP947563**				
	*Gyps coprotheres*	CV7	**OP947553**				
		CV12	**OP947562**				
		CV13	**OP947561**				
		CV17	**OP947554**				
	*Plegadis falcinellus*	GI1	**OP947555**				
		GI2	**OP947556**				
		GI3	**OP947557**				
	*Phoeniconaias minor*	LF1	**OP947558**				
		LF3	**OP947559**				
		LF4	**OP947560**				
	*Poicephalus meyeri*	MP1	**OP947564**				
	*Bubo capensis*	CEO6	**OP947567**	lCIAE02	lCIAE02	MK652257	100%
	*Phoenicopterus roseus*	GF19	**OP947568**				
	*Eudocimus ruber*	SI8	**OP947569**				
	*Bubo capensis*	CE08	**OP947565**	**lBUBCAP01**	lBUVIR02	MK947885/ EU627821	99%
*Haemoproteus*	*Bostrychia hagedash*	HI11	**OP947570**	**hBOSHAG02**	hBOSHAG01	MW546941	98%
	*Tyto alba*	BO10	**OP947572**	hTYTAL6	hTYTAL6	KU726003	100%
*Leucocytozoon*	*Tyto alba*	BO5 †	**OP947566**	lCIAE02	lCIAE02	MK652257	100%
*Haemoproteus*			**OP947571**	hTYTAL6	hTYTAL6	KU726003	100%
*Leucocytozoon*	*Tyto alba*	BO12 †	**OP947551**	lCHRKLA02	lCHRKLA02	MH492307	100%
*Haemoproteus*			**OP947573**	hTYTAL6	hTYTAL6	KU726003	100%

^†^Samples with mixed *Leucocytozoon* and *Haemoproteus* infections.

Sequences and lineages identified from this study are indicated in bold text.

*Leucocytozoon* sp. sequence lCEO8 from the Cape Eagle Owl was 99% identical to lineage lBUVIR02 from the Great Horned Owl in the United States (Fecchio et al. 2019) and was designated as a new lineage (lBUBCAP01). Two *Haemoproteus* spp. lineages are identified in this study. Lineage hTYTAL6 was originally described from owls (Pornpanom et al. 2021) and other raptors (Pornpanom et al. 2019) in Thailand and from an unnamed raptor species in China (unpublished sequence KT944102) (Figure 1), and a novel lineage from this study (hBOSHAG02) was identified from Hadada Ibis. The latter is 98% similar to lineage hBOSHAG01, also described from Hadada Ibis in South Africa (unpublished sequence, GenBank accession no. MW546941), and to lineage hCXPIP16 from the Northern House mosquito (*Culex pipiens pallens* Linnaeus, 1758) from Japan (Ejiri et al. 2011).

Two Barn Owls (BO5 and BO12) had co‐infections of *Haemoproteus* lineages hTYTAL6 and *Leucocytozoon* lineages hCIAE02 (BO5) and hCHRKLA02 (BO12). The genetic distance between lineages lBUBCAP01 and lBUVIR02 is 1%, while lineage hBOSHAG02 differs from lineages hBOSHAG01 and hCXPIP16 by 3% and 4%, respectively. The *cyt b* sequences obtained from this study have been submitted to the GenBank database (https://www.ncbi.nlm.nih.gov/genbank) with accession numbers OP947548–OP947573 and to MalAvi database.

## 4 Discussion

This is the first molecular study of haemosporidian parasites in captive birds of the orders Accipitriformes (vultures), Pelecaniformes (ibis), Psittaciformes (parrots), Phoenicopteriformes (flamingos), and Strigiformes (owls) in South Africa. We analyzed archived blood samples from the NZG animal collection. Unfortunately, blood smears were not available, and therefore, morphological studies could not be done. However, the PCR assays used in this study have been widely used to determine infection rates of avian haemosporidia in various avian hosts and in epidemiological studies where blood smears are not available. The overall infection rate of haemosporidian parasites in the study population was 36.1%. Only *Leucocytozoon* and *Haemoproteus* lineages were identified from this study, with significantly higher *Leucocytozoon* infections than *Haemoproteus* infections. Three known and two new lineages of *Leucocytozoon* and *Haemoproteus* were identified in this study.

### 4.1 Prevalence

Avian haemosporidian infections have been reported from different species of captive, non‐native birds globally, and have been associated with severe disease outbreaks in zoos globally (Graczyk et al. 1995; Richard et al. 2002; Paperna and Martelli 2008; Meister et al. 2023). Results of the current study indicate that haemosporidian parasites are circulating in the animal collection at the NZG, with an overall prevalence of at least 36.1%, dominated by *Leucocytozoon* infections. This can potentially compromise the conservation of threatened species at the national zoo if disease outbreaks occur. Taioe et al. (2020) recorded *Leucocytozoon* spp. from *Culex pipiens* and *Culex quinquefasciatus* mosquitoes at the NZG. Although mosquitoes are not biological vectors of *Leucocytozoon* parasites, *Leucocytozoon* DNA can occasionally be detected in the midgut of mosquitoes after a blood meal. However, the parasite does not develop or migrate beyond this point and is eventually digested. Haemosporidian infections at the NZG can be linked to the presence of free‐ranging birds that usually visit the zoo, and to the presence of mosquitoes and biting midges (*Culicoides* species) that have previously been reported in the zoo (Labuschagne et al. 2008; Taioe et al. 2020). Although blackflies have not been reported from the premises, the presence of *Leucocytozoon* infections from the animal collection indicates their prevalence and should be investigated. Migratory birds have also been associated with the transmission of infections to naïve bird populations in situ and ex situ (Pulgarin et al. 2018; de Angeli Dutra et al. 2021; Meister et al. 2023).

Previous studies of avian haemosporidia in South Africa indicate that these parasites commonly infect birds such as the African Penguin and other seabirds at a rehabilitation center on the west coast of South Africa, with different infection rates (0.3%–28.7%) (Parsons and Underhill 2005; Parsons et al. 2017; Botes et al. 2017), various species of waterbirds (Okanga et al. 2013; 35.7%–100%), terrestrial Afrotropical birds (Chaisi et al. 2019; 68.82%); House Sparrows (24.3%), and Southern Grey‐Headed Sparrows (60%) (Wardjomto et al. 2021), and wild passerines (1.4%–87.3%) in the Eastern Cape province (Schultz and Whittington 2005).

Although the nested PCR used in the study identified *Leucocytozoon* spp. in Cape Vultures and owls, it should be noted that the primers used in this study are sub‐optimal for determining *Leucocytozoon* spp. of raptors, especially the *Leucocytozoon toddi* complex which is the principal and most prevalent parasite in Accipitriformes (Pérez‐Rodríguez et al. 2013; Himmel et al. 2019). This limitation could have possibly underestimated infection rates in these birds. Future studies of *Leucocytozoon* infections in Accipitriformes and related orders should include specific primers for detecting these infections, such as those by Pérez‐Rodríguez et al. (2013) and Himmel et al. (2019).

### 4.2 Lineage Diversity

This study identified three *Leucocytozoon* (lCHRKLA02, lCIAE02, and lBUBCAP01) and two *Haemoproteus* lineages (hTYTAL6 and hBOSHAG02) from captive raptors and other birds at the NZG. Lineage lCHRKLA02 was the most commonly identified lineage and was identified from seven bird species (Table 2). This lineage was first reported from Klaas's Cuckoo (*Chrysococcyx klaas*) in South Africa (Chaisi et al. 2019) and was subsequently identified from *Culex* mosquitoes at the NZG by Taioe et al. (2020). Further research is required to determine the role of this lineage in the epidemiology of haemosporidian infections in South Africa and its impact on bird health as epidemics of haemosporidiosis in wild bird populations have been linked to the introduction of novel parasite lineages into naive host assemblages (Himmel et al. 2021; LaPointe et al. 2012). Although we have identified lineage lCHRKLA02 in three independent studies and samples from different localities in our lab, these results need to be verified by other labs to rule out contamination, as contaminants are a constant risk and can lead to the overestimation of host diversity for single lineages. We described the second common lineage (lCIAE02) from the Cape Eagle Owl (*Bubo capensis*), Greater flamingo (*P. roseus*), Scarlet Ibis (*E. ruber*), and from a mixed infection with lineage hTYTAL6 in one Barn Owl (*T. alba*). MalAvi records indicate that *Leucocytozoon* lineage lCIAE02 is a generalist and infects various avian species globally. It has been described from migratory, resident, and captive birds in the United Kingdom, Transcaucasia, Japan, Turkey, Poland, Spain, Russia, Sweden, Philippines, Germany, and Mongolia. Host species include the Cinereous Vulture [*Aegypius monachus* (Linnaeus, 1766)] (Krone et al. 2008), Besra [*Accipiter virgatus* (Temminck, 1822)] (Silva‐Iturriza et al. 2012), Western Marsh Harrier [*Circus aeruginosus* (Linnaeus, 1758)] (Krone et al. 2008; Harl et al. 2022), Eurasian Griffon Vulture [*Gyps fulvus* (Hablizl, 1783)] (Krone et al. 2008; Chakarov and Blanco 2021), Blackcap [*Sylvia atricapilla* (Linnaeus, 1758)] (Pérez‐Rodríguez et al. 2014), Long‐Legged Buzzard [*Buteo rufinus* (Cretzschmar, 1827)] (Ciloglu et al. 2016), Eleonora's Falcon (*Falco eleonorae* Géné, 1839) (Gutiérrez‐López et al. 2015), Caspian Gull (*Larus cachinnans* Pallas, 1811) (Zagalska‐Neubauer and Bensch 2016), European Herring Gull (*Larus argentatus* Pontoppidan, 1763) (Zagalska‐Neubauer and Bensch 2016), Long‐Eared Owl [*Asio otus* (Linnaeus, 1758)] (Ciloglu et al. 2016), Eurasian Eagle‐Owl [*Bubo bubo* (Linnaeus, 1758)] (Huang et al. 2020), Egyptian Vulture [*Neophron percnopterus* (Linnaeus, 1758)] (Chakarov and Blanco 2021), Eastern Imperial Eagle (*Aquila heliaca* Savigny, 1809) (Lertwatcharasarakul et al. 2021), and others. In South Africa, Chaisi et al. (2019) identified this lineage from the Woodland Kingfisher [*Halcyon senegalensis* (Linnaeus, 1766)] and Diederik Cuckoo [*Chrysococcyx caprius* (Boddaert, 1783)]. The third *Leucocytozoon* lineage, lBUVIR02, was first described by Fecchio et al. (2019) in the Great Horned Owl [*Bubo virginianus* (Gmelin 1788)] in the United States and is a common lineage in this host species.

Two novel lineages (lBUBCAP01 and hBOSHAG02) were described from the Hadada Ibis (*B. hagedash*) and Barn Owl (*T. alba*) in this study, while the *Haemoproteus* lineage hTYTAL6 was identified from three Barn Owls, with two of these birds harboring mixed infections with *Leucocytozoon* spp. *Haemoproteus* lineage hTYTAL6 was originally reported from Barn Owls at a raptor rehabilitation center in Thailand by Pornpanom et al. (2019). So far, hTYTAL6 together with others (hTYTAL3, hTYTAL4, hTYTAL5, and hTYTAL7) (Pornpanom et al. 2019) has only been reported in raptors. Barn Owls share lineages pLINN1, lCIAE02, and lCHRKLA02 with bird species of other orders. Our analyses also support the observation by Pornpanom et al. (2019) that *Haemoproteus* sequences of owls from Africa, Asia, Europe, and North America are phylogenetically related, indicating a common ancestor.

The absence of *Plasmodium* lineages in the study was unexpected as *Plasmodium* spp. have been identified from mosquitoes (*Culex* spp.) (Taioe et al. 2020) and from live and dead penguins (unpublished) at the zoo. This result could be due to the decreased sensitivity of the PCR used in this study in detecting *Plasmodium* spp. in mixed infections, as observed by Bernotiene et al. (2016) and others. Alternative primers such as those used by Beadell et al. (2004), a combination of various primer sets as recommended (Martinsen et al. 2008; Beadell et al. 2009; Bernotiene et al. 2016), or genus‐ or species‐specific primers (Martinsen et al. 2006; Pérez‐Rodríguez et al. 2013; Ciloglu et al. 2019; Himmel et al. 2019) are recommended to fully explore avian haemosporidian diversity in similar studies.

### 4.3 New Locality and Host Ranges

New host records in the MalAvi database from this study include parasites of the Cape Parrot (*Poicephalus robustus*), Cape Vulture (*G. coprotheres*), Hadada Ibis (*B. hagedash*), Glossy Ibis (*Plegadis falcinellus*), and Scarlet Ibis (*E. ruber*). There is only one other record of haemosporidian parasites of Meyer's Parrot (*Poicephalus meyeri*) from Malawi that was infected by *Plasmodium* lineage pNI02 (Lutz et al. 2015). Phoenicopteriformes are also an understudied group of birds, with a few parasite records from the Lesser Flamingo (*Phoeniconaias minor*), Greater Flamingo (*P. roseus*), and the Chilean Flamingo (*Phoenicopterus chilensis*) from South Africa (this study), Switzerland (Meister et al. 2023), Texas (Ferrell et al. 2007), and Brazil (Chagas et al. 2017). This highlights the need for more surveys of avian haemosporidia in understudied bird species such as flamingos, as they are possible reservoir hosts of these parasites.

In conclusion, this study indicates that captive birds at the NZG have high infection rates of haemosporidia and are a source of infections to insect vectors and susceptible hosts at the NZG and other facilities where the birds are translocated for conservation or trade. It also increases our knowledge of new geographic and host ranges of haemosporidian parasites of raptors and other understudied bird species and highlights the importance of wildlife biobanks in studies of disease dynamics in birds and other captive animals, especially for threatened taxa where the collection of samples is restricted and therefore information on host–pathogen disease dynamics is lacking. Vectors of the parasites identified in this study are unknown; however, transmission might be attributed to simulium flies and biting midges that are common in the zoo. Although most bird species are not severely affected by blood parasites, strict biosecurity measures and regular surveillance and monitoring of infections both in situ and ex situ may be required to prevent disease outbreaks in the animal collection that might compromise conservation efforts of vulnerable and threatened species. Subsequent studies, especially from field samples, are recommended to describe the observed lineages to the species level and for a more comprehensive analysis of the prevalence and impact of these parasites on avian health.
